# The effects of high-quality nursing accompaniment on the sleep quality, negative psychological moods and quality of life of patients with acute myocardial infarct

**DOI:** 10.3389/fcvm.2025.1562236

**Published:** 2025-07-08

**Authors:** Hongdi Zhou, Jing Zhang, Yaqiang Li

**Affiliations:** ^1^ Department of Cardiovascular Medicine, Yingshang County People’s Hospital, Fuyang, China; ^2^ Department of Neurology, People’s Hospital of Lixin County, Bozhou, China

**Keywords:** acute myocardial infarct, high-quality nursing, accompaniment, psychological state, Pittsburgh sleep quality index, self-rating anxiety scale, self-rating depression scale, generic quality of life inventory-74

## Abstract

**Objectives:**

High-quality nursing accompaniment serves as a paragon of patient-centered care, distinguished by the provision of exceptional nursing services that integrate thorough physical care with robust psychological support. The present study aimed to investigate the effects of high-quality nursing accompaniment intervention on sleep quality, negative psychological moods, and quality of life in patients with acute myocardial infarct (AMI).

**Methods:**

This study recruited patients with AMI from January 2023 to December 2024. Patients were divided into two cohorts: an observation group and a control group. The control group received standard conventional nursing care, while the observation group underwent high-quality nursing accompaniment interventions. The two groups were compared with respect to sleep quality, psychological status and quality of life, as well as complications, before and after intervention. The two groups were compared with respect to sleep quality [Pittsburgh Sleep Quality Index (PSQI)], psychological status [self-rating anxiety scale [SAS] and self-rating depression scale [SDS] scores], and quality of life [generic quality of life inventory-74 (GQOLI-74) scores], as well as satisfaction and complications, before and after intervention.

**Results:**

The results indicated that the observation group exhibited superior outcomes compared to the control group following the intervention, as evidenced by reduced PSQI scores. Post-intervention, both groups experienced a decline in the scores for the SAS and the SDS, with a notably greater reduction observed in the observation group (*P* < 0.05). Furthermore, after the intervention, improvements were noted in all dimensions of the GQOLI-74 for both groups, yet the observation group demonstrated a more pronounced enhancement (*P* < 0.05). Additionally, the observation group reported a lower incidence of complications and higher levels of nursing satisfaction.

**Conclusions:**

High-quality nursing accompaniment can effectively improve the sleep quality, alleviate adverse psychological conditions, elevate overall quality of life. This approach is associated with a minimal rate of complications and a high level of nursing satisfaction.

## 1 Introduction

Cardiovascular disease (CVD) ranks as one of the foremost causes of mortality in community settings worldwide, resulting in approximately 17.9 million deaths each year. Notably, coronary heart disease alone accounts for nearly half of these fatalities (1). Acute myocardial infarction (AMI) is a critical and common cardiac event, characterized by the sudden cessation of blood flow to the myocardium, which leads to tissue necrosis. Atherosclerotic plaque rupture within the coronary arteries is a major cause of AMI. This rupture is often triggered by several risk factors, including hypertension, hyperlipidemia, diabetes mellitus, and tobacco use (2, 3). The pathophysiological mechanisms underlying AMI encompass thrombosis, inflammation, and subsequent ischemic injury. These processes collectively contribute to the high morbidity and mortality rates associated with AMI on a global scale. Annually, the incidence rate is estimated to be approximately 80–100 cases per 100,000 individuals (4). The global burden of disease has shown that high systolic blood pressure (54.6%), elevated low-density lipoprotein cholesterol (46.6%), and smoking (23.9%) are the three leading contributors to the disability-adjusted life years (DALYs) lost due to ischemic heart disease (5). The impact of AMI extends beyond the physical realm. Numerous studies have shown that patients often experience poor sleep quality, heightened emotional distress, and a significant reduction in their overall quality of life following the event (6–10). Sleep disturbances can exacerbate cardiac conditions and hinder recovery, while psychological factors, particularly anxiety and depression, are prevalent among AMI patients, significantly affecting their emotional well-being (11). Furthermore, individuals often report a lower quality of life due to lifestyle modifications and ongoing health concerns following AMI. This multifaceted impact on sleep, emotional health, and quality of life underscores the need for comprehensive care approaches tailored to the psychological and psychosocial dimensions of recovery. Traditional nursing care methods primarily emphasize the acute management of AMI by focusing on physiological stabilization and medical interventions. However, these approaches often fail to address the comprehensive needs of patients, particularly their emotional and psychological well-being. To enhance recovery outcomes, there is a critical need to develop integrative care models that incorporate mental health support and lifestyle modifications alongside standard cardiac rehabilitation practices. By prioritizing both physical and psychological health in the care of individuals recovering from AMI, healthcare providers can significantly improve their overall quality of life.

Accompaniment can be viewed as a comprehensive social support strategy that encompasses practical assistance (e.g., transportation), emotional encouragement (e.g., moral support), and communication facilitation (e.g., enhancing patient–provider interactions), all of which have been shown to impact cardiovascular outcomes (12, 13). High-quality nursing represents an exemplary model of patient-centered care, characterized by the delivery of superior nursing experiences that encompass both physical care and psychological support. This approach not only highlights the compassionate aspects of nursing accompaniment but also enhances the overall comprehensiveness quality of nursing services provided to patients (14). Furthermore, high-quality nursing accompaniment interventions are guided by the principles of holistic nursing care, thereby reinforcing the core aspects of clinical nursing services. This approach is characterized by its systematic and comprehensive nature, making it highly specific in its application. This specific nursing framework has been extensively utilized in a range of specialized fields, such as neurology and hematology, and has produced positive results. Research carried out by Xiao et al. demonstrates that providing high-quality nursing care to elderly patients suffering from hypertension can notably alleviate their negative emotional states and enhance their overall quality of life, while also resulting in a reduced occurrence of complications (15). Furthermore, the provision of high-quality nursing accompaniment for elderly individuals suffering from acute cerebral infarction significantly improves neurological recovery, reduces negative psychological conditions, and enhances overall quality of life. This intervention is also linked to a decrease in complication rates and an increase in nursing satisfaction, thereby validating its clinical significance (16). Moreover, a study conducted by Zuo et al. emphasizes that the implementation of high-quality nursing interventions yields numerous advantages in the management of elderly patients undergoing cataract surgery. These benefits include a significant reduction in intraocular pressure, an improvement in the patients’ overall quality of life, decreased occurrence of postoperative complications, and an increase in patient satisfaction levels (17).

Despite the potential benefits of high-quality nursing accompaniment, empirical research on its application among patients with acute AMI remains limited. Therefore, this study aims to implement high-quality nursing accompaniment interventions for patients with AMI and to evaluate their effects on sleep quality, negative psychological states, and overall quality of life. By doing so, we hope to demonstrate the feasibility and effectiveness of this nursing model as a potential intervention program.

## 2 Materials and methods

### 2.1 Subjects

#### 2.1.1 Data collection and assessment

The present prospective observational analysis recruited all participants who were subsequently diagnosed with AMI at Yingshang County People's Hospital from January 2023 to December 2024. This prospective cohort study initially enrolled 180 consecutive patients diagnosed with AMI. The participants were randomly assigned to either the control group (*n* = 90) or the intervention group (*n* = 90) through a computer-generated randomization sequence. The study protocol received approval from the Institutional Review Board of Yingshang County People's Hospital, and written informed consent was obtained from all conscious participants or their legally authorized representatives using standardized proxy consent forms.

**Figure 1:**
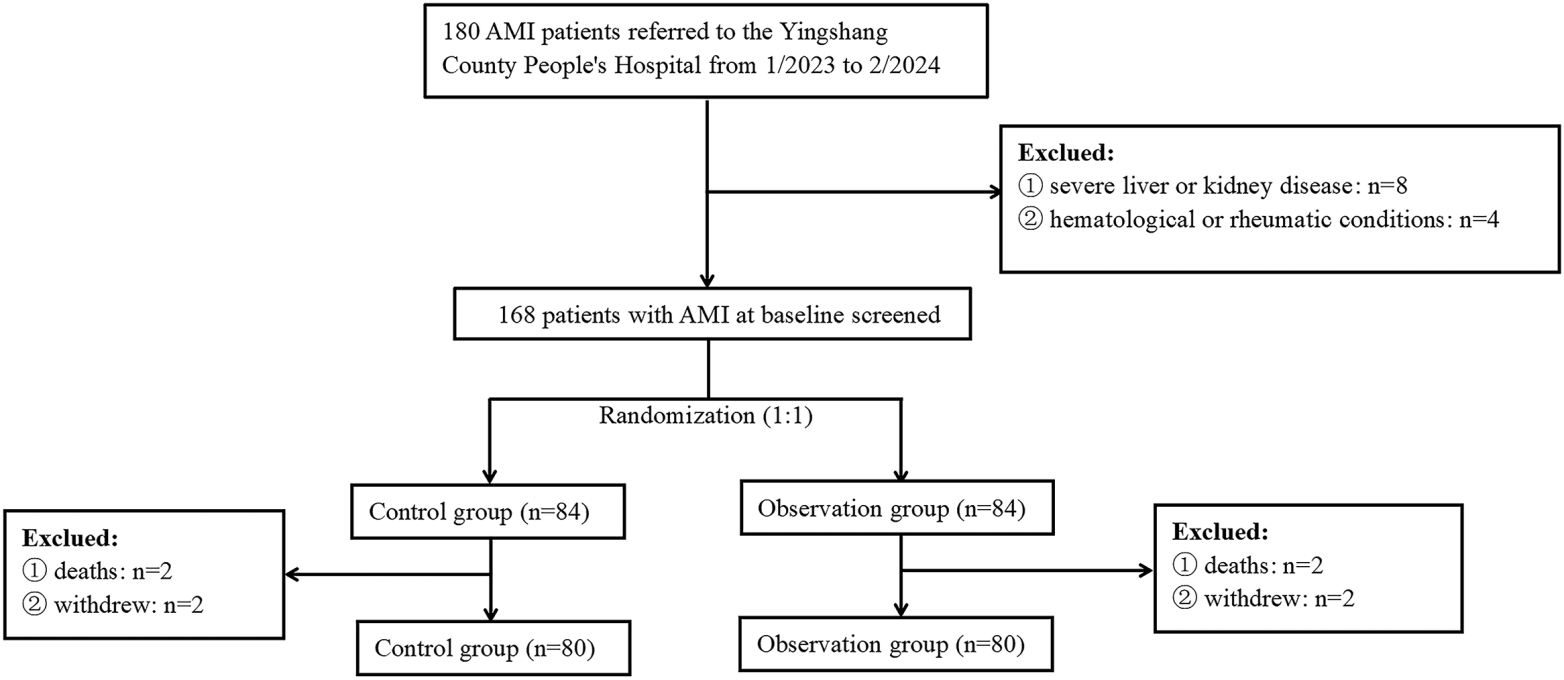
Flowchart of participant selection. AMI, acute myocardial infarction.

The research adhered strictly to the ethical principles outlined in the Declaration of Helsinki. Patients were excluded from the study if they met any of the following criteria: (1) severe consciousness disorder; (2) presence of multiple serious comorbidities, including severe liver or kidney disease, autoimmune disorders, hematological or rheumatic conditions, and malignant tumors; or (3) inability to attend follow-up visits or having missed follow-up visits. Participants included in the study were required to meet specific criteria: they had to be aged between 18 and 80 years, and all participants had to fulfill the clinical diagnostic criteria for AMI, with their diagnoses confirmed through electrocardiography (ECG). During the study period, a total of 180 patients meeting the diagnostic criteria for AMI were initially admitted to the hospital. Among them, 8 patients with severe liver or kidney disease, 4 with hematological or rheumatic conditions, and 4 who died within the first 3 months of follow-up were excluded. Additionally, 4 patients withdrew from the study after discharge. Consequently, 160 patients were ultimately included in the study, with 80 in the observation group and 80 in the control group (Figure 1).

### 2.2 Conventional nursing

In the control group, patients were received routine nursing intervention, which included dietary instructions, bed rest, and basic nursing measures such as monitoring, oxygenation, and prevention of complications. During the acute phase, which is defined as the first 48 h after onset, we observed that the control group had an average of 10–15 contacts per day. In contrast, the observation group, which benefited from high-quality nursing accompaniment, experienced a significantly higher average of 20–30 contacts per day. As the patients transitioned into the recovery phase, the observation group maintained an average of 6–8 contacts per day, while the control group had slightly fewer, averaging 3–4 contacts per day.

### 2.3 High-quality nursing accompaniment

In addition to the basic treatment for the control group, the research group was provided with high-quality nursing accompaniment. (1) Construct a high-quality nursing accompaniment team: Nursing staff with extensive work experience and a high level of responsibility for their duties were selected in order to form a specialised quality nursing team, which was headed by a nurse supervisor and the team members were responsibility nurses. (2) Provision of comprehensive health education: Nursing staff should provide patients and their families with relevant information about acute myocardial infarction, so that they can understand the causes of the disease, treatment methods, nursing measures and prognosis, which on the one hand helps to enhance patients’ understanding of their own condition and on the other hand can reduce their psychological pressure. (3) Psychological nursing: Affected by the physical discomfort caused by the disease, and worry about their own condition and other factors, patients will feel anxious, fearful, and frustrated. Nursing staff should establish a good relationship of trust with the patient, so that the patient feels a sense of security and trust, and fully understand their psychological condition, care for and love them, and provide psychological support. In addition, the nursing staff understood the psychological problems of the patients by communicating with them and helped them to solve their psychological problems through psychological counseling, psychological suggestions, and relaxation training. (4) Provision of high-quality medical environment: Strict adherence to hospital infection control norms is essential for maintaining a high standard of cleanliness and disinfection in patient wards. It is important to ensure that these areas are not only clean but also meet disinfection qualifications. Additionally, proper regulation of temperature and humidity is crucial; typically, the temperature should be maintained between 22 and 24°C, while humidity levels should range from 50% to 60%. Furthermore, it is advisable to regularly open the windows of patient wards to facilitate ventilation, thereby enhancing both the physical and mental comfort of patients. (5) Sleeping nursing: Create a quiet, warm, cool, and dry sleeping environment for patients, which facilitates their ability to fall asleep. Additionally, assist patients in developing good work and rest habits, such as maintaining regular activities throughout the day and ensuring sufficient rest at night to align with their physiological rhythms. If the patient experiences difficulty falling asleep, it is advisable to engage in quiet activities, such as reading or listening to soft music, 1–2 h before bedtime. These activities can facilitate relaxation and promote the onset of sleep. It is essential to avoid caffeinated beverages, including coffee, tea, and cola, as well as to eliminate stimulating behaviors like smoking and drinking alcohol. In addition, according to the patient's condition and physical status, the patient is instructed to adopt appropriate positions, such as a semi-recumbent position, or a left lateral position, etc., in order to promote better sleep. Prior to bedtime, patients are advised to immerse their feet in warm water and consume hot milk. Additionally, it is beneficial to play gentle music in the patient's room and provide items such as grapefruits to enhance the sleep-promoting environment. It is essential to meticulously monitor and document the patient's sleep patterns, particularly to identify any significant sleep disorders. Any findings should be promptly communicated to the physician. Additionally, the administration of sleep medications should be carried out in accordance with the physician's guidance. (6) Pain nursing: Consistent evaluation of patients’ pain is essential for comprehending both the intensity and nature of their discomfort. For patients experiencing mild pain, non-pharmacological strategies such as engaging in conversation or listening to music may be utilized to distract from the sensation of pain, thereby alleviating their discomfort. On the other hand, in cases where pain is more severe, it is imperative to administer appropriate analgesics in accordance with the physician's directives. (7) Dietary nursing: Individuals suffering from acute myocardial infarction often exhibit a deterioration in their condition following a substantial meal, particularly one that is high in fat content. Symptoms such as recurrent vomiting, abdominal distension, indigestion, excessive gas, or even diarrhea may occur, potentially culminating in the risk of sudden death subsequent to a bowel movement. Consequently, regarding dietary practices, one may undertake fasting judiciously or initially implement a liquid diet, progressively transitioning to a more substantial intake. The diet should primarily consist of light, easily digestible foods while simultaneously adhering to low salt and low fat guidelines. Patients who have experienced an acute myocardial infarction are typically prescribed strict bed rest, which may lead to constipation. Consequently, it is advisable to encourage these patients to consume foods high in fiber, such as leafy green vegetables, legumes, whole grains, and low-sugar fruits. Such dietary choices can help slow down the absorption of food, attenuate the postprandial spikes in blood glucose levels, and thereby assist in rectifying disturbances in glucose and lipid metabolism. Additionally, these foods promote intestinal motility, which is instrumental in preventing constipation. Furthermore, they support the intake of essential vitamins and trace minerals. (8) Moderate exercise: When the patient's condition is stable and the cardiac function is adequate, the patient will be instructed to gradually start exercise rehabilitation training, with aerobic exercise as the main focus. The intensity and frequency of exercise will be reasonably adjusted according to the patient's condition, ensuring that the patient does not feel fatigue. (9) Post-Discharge Nursing Accompaniment: Nursing staff tailor and execute regular follow-up plans based on the patient's condition, closely monitoring changes in the patient's health and the specifics of home care implementation at each stage. They then adjust and update the patient's recovery plan accordingly. The forms of regular follow-up include home visits, patient return visits to the hospital, telephone follow-ups, online follow-ups, or written inquiries. (10) Family involvement in accompanying patients through treatment and rehabilitation: Nursing staff play a crucial role in guiding patients to adopt healthier lifestyle habits, such as quitting smoking and reducing alcohol consumption. They recommend a balanced diet that is low in salt and fat, which is essential for preventing coronary heart disease. In addition to following a comprehensive prevention plan, nurses encourage patients to engage in suitable exercise routines and adhere to their prescribed medications. They also focus on enhancing the understanding of the disease among patients and their families, fostering an environment where both patients and their loved ones are actively involved in the treatment and rehabilitation process. This collaborative approach not only helps to improve patients’ knowledge but also boosts their confidence in managing their health and adhering to treatment protocols. The intervention effect was assessed 3 months later.

### 2.4 Clinical data collection

Baseline characteristics include demographic data (including age and gender) were collected for patients at admission. Venous blood was drawn from patients the next morning after admission. We measured white blood cell (WBC) counts, glucose(G), triglyceride(TC), triglycerides(TG), high-density lipoprotein (HDL), low-density lipoprotein (LDL), apolipoprotein A (ApoA), and apolipoprotein B (ApoB).

### 2.5 Observation indicators

Patients were asked to return to the hospital for a follow-up appointment three months post-intervention. The pertinent evaluation scales were administered to the patients prior to the intervention (upon admission) and subsequently three months post-onset. In instances where patients were unable to attend follow-up appointments as scheduled, nursing staff facilitated the completion of the forms via follow-up telephone calls.

(1) **Sleep quality:** The evaluation of sleep quality was conducted using the Pittsburgh Sleep Quality Index (PSQI), which encompasses various factors such as overall sleep quality, sleep efficiency, use of hypnotic medications, total sleep duration, presence of sleep disturbances, time required to initiate sleep, and daytime functioning. Each component was assigned a score ranging from 0 to 3, where a lower score indicates an enhanced quality of life. Higher quality of sleep (18).
(2) **Psychological parameters:** the assessment of anxiety and depression in patients was conducted utilizing the Self-rating Anxiety Scale (SAS) and the Self-rating Depression Scale (SDS) (19). As the scores ascended, there was a corresponding rise in the levels of anxiety and depression. The two assessment tools comprised 20 items each, evaluated on a four-point scale that spanned from 0 to 100, where lower scores were indicative of a more favorable psychological condition.
(3) **Quality of Life Metrics:** The scores of the Generic Quality of Life Inventory-74 (GQOLI-74) were evaluated before and after a three-month intervention in both groups. This instrument encompasses four dimensions: material life, physical function, psychological function, and social function. The scoring range for the physical function dimension is from 16 to 80, while for the other three dimensions, it spans from 20 to 100. Higher scores correspond to an improved quality of life for the patient (20).
(4) **Patient Satisfaction**: A customized “Patient Clinical Care Satisfaction Questionnaire” was employed to evaluate the degree of patient satisfaction following the intervention in both groups. This assessment instrument consists of five items, allowing for a total score that can reach 100. A higher score indicates increased satisfaction regarding the nursing care provided. Furthermore, occurrences of clinical complications following the intervention were carefully recorded and analyzed comparatively between the groups. Furthermore, occurrences of clinical complications following the intervention were carefully recorded and analyzed through inter-group comparisons (21).

### 2.6 The approach to documenting intervention measures

In the manuscript, we have included a comprehensive description of the methods used to document the intervention. To ensure precise recording and verification of the intervention process, we implemented several key measures.

(1) **Intervention Process Recording**: The intervention process for each patient was carefully recorded by the assigned nurse using a specially designed intervention record form. This form included comprehensive details, such as the precise timing of each intervention session, the content provided during the session, the duration of the intervention, and feedback from the patient.
(2) **Regular Supervision and Verification**: Our research team carried out weekly assessments of the intervention record forms to verify their completeness and accuracy. Furthermore, we maintained regular communication with patients to ensure that the interventions were being implemented as intended.
(3) **Data Management**: All intervention records were systematically stored in an electronic database, facilitating easy access and analysis. Additionally, any deviations or issues that arose during the intervention process were meticulously documented and analyzed to ensure the smooth implementation of the interventions.

### 2.7 Statistical analysis

Statistical analyses were conducted utilizing the R statistical software (version 4.4.3; R Foundation for Statistical Computing, Vienna, Austria). Continuous variables were summarized using the mean ± standard deviation (SD) or the median with the interquartile range (IQR). In contrast, categorical variables were represented as frequencies with corresponding percentages. To evaluate the differences in baseline characteristics across the groups, independent sample *t*-tests were utilized for continuous variables that met parametric assumptions, while the Mann–Whitney *U* tests were employed for non-parametric continuous variables. For categorical variables, the chi-squared test or Fisher's exact test was applied, depending on the suitability of the data. A *p*-value of less than 0.05 was considered indicative of statistical significance.

## 3 Results

### 3.1 Clinical and demographic characteristics of patients in observation group and control group

The study consisted of 160 participants in total, with 80 in the control group (46 males) and 80 in the observation group (57 males). The mean age of the control group was noted to be 63.52 ± 10.42 years, in contrast to the observation group, which exhibited a mean age of 63.31 ± 10.23 years. This suggests that there was no statistically significant difference between the two groups (*P* > 0.05). Moreover, the other baseline characteristics revealed no significant disparities among the participant groups, as presented in Table 1.

**Table 1:** Clinical and demographic characteristics of patients in observation group and control group.

Variables	Observation group (*N* = 80)	Control group (*N* = 80)	Z/t/*χ*^2^ value	*P* value
Demographic characteristics				
Gender, males, *n* (%)	57 (71.25)	46 (57.50)	3.30	0.069
Age, years, mean ± SD	63.31 ± 10.23	63.52 ± 10.42	−0.478	0.802
Laboratory findings (IQR)				
WBC, ×10^9 ^/L, median (IQR)	6.22 (5.18–7.54)	6.52 (5.63–7.51)	−0.985	0.334
Glucose, mmol/L, median (IQR)	5.23 (4.67–7.51)	5.24 (4.67–6.86)	−1.128	0.242
TG, mmol/L, median (IQR)	1.32 (0.79–1.79)	1.14 (0.56–1.97)	0.276	0.873
TC, mmol/L, median (IQR)	4.25 (3.54–5.76)	4.34 (3.65–5.44)	−0.498	0.632
HDL-C, mmol/L, median (IQR)	1.21 (0.67–1.39)	1.13 (0.79–1.46)	0.287	0.854
LDL-C, mmol/L, median (IQR)	2.34 (2.09–3.56)	2.35 (1.78–3.43)	1.134	0.234
ApoA, g/L, median (IQR)	1.24 (1.23–1.65)	1.29 (1.12–1.87)	−1.204	0.224
ApoB, g/L, median (IQR)	0.78 (0.56–1.32)	0.88 (0.67–1.23)	−0.674	0.498

WBC, white blood cell; G, glucose; TC, triglyceride; TG, triglycerides; HDL, high-density lipoprotein; LDL, low-density lipoprotein; ApoA, apolipoprotein A; ApoB, apolipoprotein B.

### 3.2 The PSQI scores between the two groups of patients

Prior to the intervention, no statistically significant differences were observed in the Pittsburgh Sleep Quality Index (PSQI) scores between the two groups (all *P* > 0.05). Following the nursing intervention, a reduction in PSQI scores was noted in both groups, with the intervention group exhibiting significantly lower scores compared to the comparison group (all *P* < 0.05), as illustrated in Table 2.

**Table 2:** Comparison of PSQI scores before and after intervention.

Variables	Observation group (*N* = 80)		Control group (*N* = 80)	
	Before intervention	After intervention	Before intervention	After intervention
Sleep quality	2.23 ± 0.34	1.16 ± 0.23*,**	2.22 ± 0.32	1.81 ± 0.23*
Sleep efficiency	2.42 ± 0.33	1.14 ± 0.12*,**	2.49 ± 0.37	1.99 ± 0.26*
Hypnotic drugs	2.48 ± 0.32	1.34 ± 0.31*,**	2.48 ± 0.27	1.94 ± 0.28*
Sleep time	2.52 ± 0.33	1.32 ± 0.16*,**	2.54 ± 0.33	2.03 ± 0.24*
Sleep disorder	2.10 ± 0.24	1.02 ± 0.05*,**	2.16 ± 0.33	1.68 ± 0.07*
Time to fall asleep	2.25 ± 0.45	1.02 ± 0.23*,**	2.23 ± 0.23	1.78 ± 0.12*
Daytime function	2.44 ± 0.23	1.30 ± 0.12*,**	2.44 ± 0.33	1.86 ± 0.14*

PSQI, Pittsburgh sleep quality index. Compared with before the nursing, *P < 0.05, compared with the control group, **P < 0.05.

### 3.3 The SAS and SDS scores between the two groups of patients

**Figure 2:**
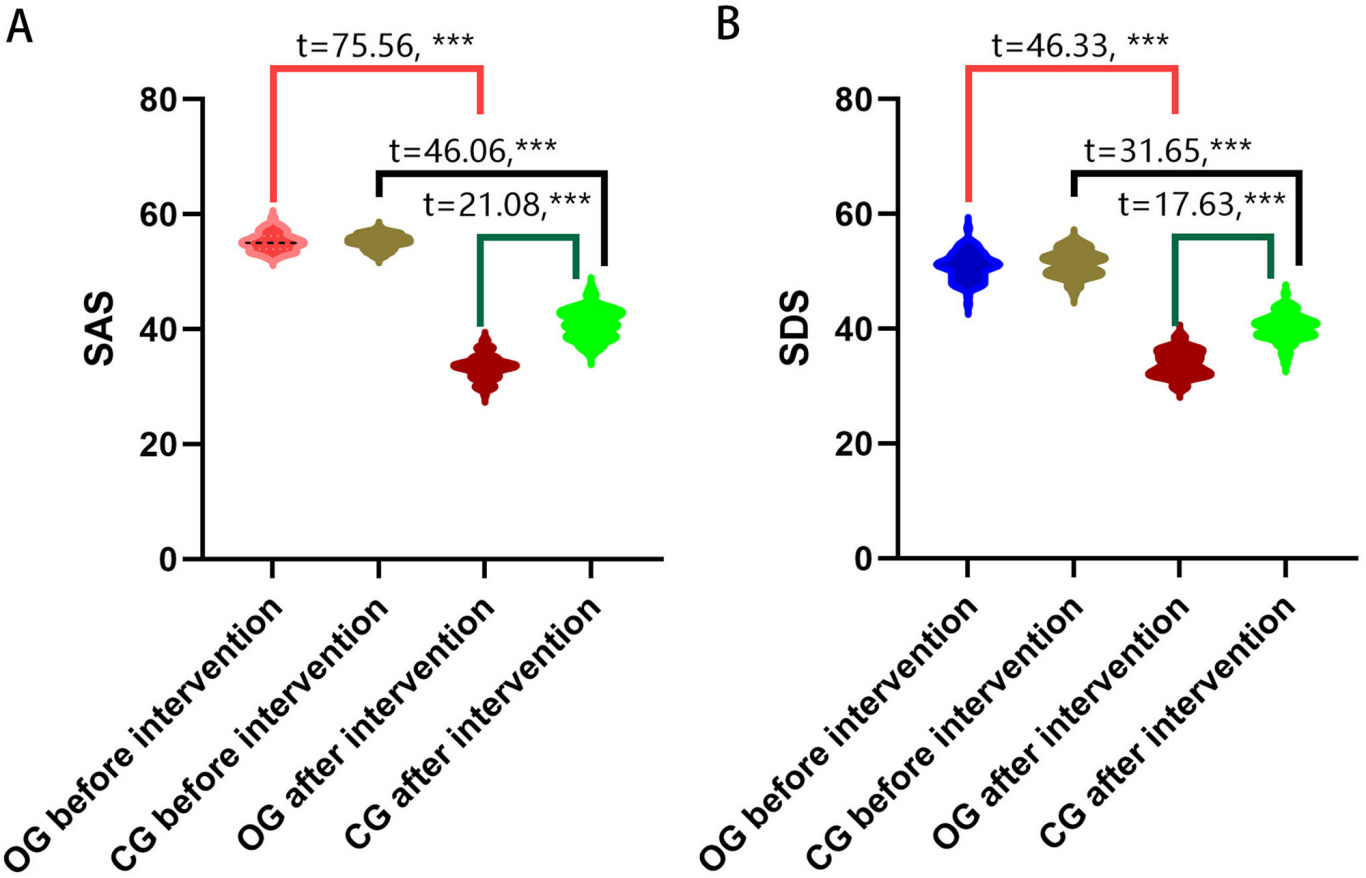
Comparison of the SAS and SDS scores. **(A)** SAS scores of the two groups before and after intervention; **(B)** SDS scores of the two groups before and after intervention. OG, observation group; CG, control group; SAS, self-rating anxiety scale; SDS, self-rating depression scale; ***P < 0.001.

Negative emotions were evaluated in both groups before and after the intervention using the SAS scale and SDS scale. The preliminary assessment revealed no statistically significant differences in the scores of the SAS and SDS between the two groups (*P* > 0.05). Following the intervention, both groups showed significant improvements in SAS and SDS scores, and the observational group demonstrated an even greater improvement (*P* < 0.05) (Figure 2).

### 3.4 Comparison of GQOLI-74 scores between the Two groups

Before the intervention, there were no statistically significant differences in scores across the four dimensions of social functioning, physical functioning, mental functioning, and material life between the observation and control groups (*P* > 0.05). After the intervention, the scores on all dimensions of the GQOLI-74 were improved in both groups, but the observation group had higher scores (all *P* < 0.05; Table 3).

**Table 3:** Comparison of GQOLI-74 scores before and after intervention.

Time	Group	Social function	Physical function	Psychological function	Material life
Before intervention	Observation group (*n* = 80)	62.44 ± 2.98	64.51 ± 2.76	62.12 ± 2.67	60.37 ± 3.65
	Control group (*n* = 80)	62.29 ± 2.89	64.21 ± 2.81	62.08 ± 2.84	60.21 ± 3.42
	t-value	0.612	0.687	0.432	0.411
	*P* value	0.412	0.342	0.512	0.365
After intervention	Observation group (*n* = 80)	87.53 ± 3.11	82.32 ± 3.13	88.32 ± 3.01	86.42 ± 2.21
	Control group (*n* = 80)	71.91 ± 2.87	70.33 ± 2.98	70.31 ± 2.12	72.12 ± 3.12
	t-value	21.112	30.121	25.123	23.431
	*P* value	0.000	0.000	0.000	0.000

GQOLI-74, generic quality of life inventory-74.

### 3.5 Comparison of complication rates between the two groups

At the three-month follow-up evaluation, the observation group documented two notable complications, both classified as arrhythmias. Conversely, the control group encountered a total of six arrhythmias, in addition to two occurrences of cardiogenic shock, two instances of acute heart failure, two cases of stroke, and one reported fatality. The results indicate that the total incidence of complications in the observation group was significantly lower than that in the control group (*P* < 0.05, as illustrated in Table 4).

**Table 4:** Comparison of complication rates between the Two groups.

Group	Observation group (*N* = 80)	Control group (*N* = 80)	*χ*^2^ value	*P* value
Arrhythmia	2 (2.5)	6 (7.5)	–	–
Cardiogenic shock	0 (0)	2 (2.5)	–	–
Acute heart failure	0 (0)	2 (2.5)	–	–
Stroke	0 (0)	2 (2.5)	–	–
Death	0 (0)	1 (1.25)	–	–
Total incidence	2 (2.5)	13 (16.25)	8.901	0.003

### 3.6 Comparison of nursing satisfaction between the two groups

In the observation group, a total of 55 patients expressed a high level of satisfaction with the nursing care they received, with 24 indicating satisfaction and only 1 reporting dissatisfaction. This resulted in an impressive nursing satisfaction rate of 98.75%. Conversely, in the control group, 50 patients reported being very satisfied with the nursing care, while 23 were satisfied and 7 expressed dissatisfaction, leading to a nursing satisfaction rate of 91.25%. A noteworthy finding was that the nursing satisfaction rate among patients in the observation group was considerably higher than that found in the control group. The statistical analysis confirmed that this difference was significant (*P* < 0.05, Table 5).

**Table 5:** Comparison of the nursing satisfaction [*n* (%)].

Group	Observation group (*n* = 80)	Control group (*n* = 80)	*χ*^2^ value	*P* value
Very satisfied	55 (68.75)	50 (62.5)	–	–
Satisfied	24 (30)	23 (28.75)	–	–
Dissatisfied	1 (1.25)	7 (8.75)	–	–
Nursing satisfaction	79 (98.75)	73 (91.25)	4.737	0.030

## 4 Discussion

This study pioneers the exploration of high-quality nursing accompaniment for patients with AMI, with a focus on its impact on sleep quality, negative psychological states, and overall quality of life. AMI is a prevalent and severe cardiovascular condition that poses a significant threat to patients’ lives. The disease predominantly affects middle-aged and elderly individuals. Given that the majority of patients are elderly, their physical capabilities tend to decline progressively with age, which can diminish the efficacy of healthcare interventions. Therefore, clinical care for this particular patient group is particularly important, which has also prompted leading medical institutions to actively reform nursing management. As a result, clinical nursing care for this patient population has garnered significant attention. In parallel with the rapid growth of China's economy, there has been a marked increase in the public demand for health services. Patients today seek not only effective treatment outcomes but also a comfortable and supportive experience throughout their care journey. Recognizing the importance of this shift, leading medical institutions are actively engaged in reforming nursing management to enhance both the quality and efficiency of nursing services, thereby improving the overall patient experience.

The PSQI score in the observation group after the intervention was lower than that in the control group, demonstrating that high-quality nursing accompaniment significantly improved sleep quality in patients with AMI. The probable reason for this is that high-quality nursing accompaniment can enhance the quality of patients’ sleep in several aspects. Firstly, high-quality nursing accompaniment emphasizes addressing the psychological needs of patients. Through psychological counseling, suggestion, and other therapeutic methods, it aids in alleviating anxiety, fear, and other negative emotions. These emotional states can significantly impact sleep quality; therefore, improving patients’ negative emotions can lead to corresponding enhancements in their sleep quality. Secondly, high-quality nursing accompaniment focuses on creating a comfortable ward environment for patients by minimizing noise and distractions, promoting better sleep. Thirdly, to improve sleep quality, nursing staff guided and encouraged patients to establish consistent sleep routines through high-quality nursing accompaniment practices. Finally, nursing staff will instruct patients to adopt appropriate lying positions, such as the semi-recumbent position or the left lateral position, according to their conditions and physical status. This can not only effectively improve the quality of sleep but also reduce the burden on the heart. In their report, Li et al. demonstrate that high-quality nursing accompaniment interventions effectively improve the quality of life (QOL), sleep quality, and nursing satisfaction in nasopharyngeal carcinoma patients undergoing radiotherapy (22). Similarly, Jiang et al. demonstrated that after high-quality nursing intervention, the PSQI scores of patients in the two groups decreased markedly, and the scores in the research group were significantly lower than those in the control group (23).

Negative emotions, such as anxiety, depression, tension, and pain, occur when individuals face situations or events that do not meet their expectations (24). A review has demonstrated that women are significantly more likely than men to experience depression and anxiety after an acute myocardial infarction, which is associated with increased morbidity, rehospitalization, and mortality, along with decreased quality of life (8). The results of our study indicated that the scores for the SAS scale and SDS scale in the observation group were markedly reduced compared to those in the control group after the intervention. This finding implies that high-quality nursing accompaniment is effective in alleviating adverse psychological states in patients with AMI. Firstly, the probable reason for this is that in conventional nursing, the nursing staff did not pay attention to the changes in the psychological state of the patients and did not provide them with targeted psychological care and related interventions, thus making it difficult to improve their negative emotions. Secondly, the use of high-quality nursing accompaniment to convey information about the disease to the patient helps to increase the patient's awareness of their disease, which can enhance the patient's sense of control over the disease, thereby helping to reduce their negative emotions, such as anxiety and depression (25). Thirdly, nursing staff help to improve the quality of their sleep by guiding and encouraging patients to develop a regular routine, thus enhancing the quality of their sleep. Finally, the responsible nurse instructs the patient to adopt an appropriate lying position, such as a semi-recumbent position or the left lateral position, according to the patient's condition and physical status. This can not only effectively improve the patient's sleep quality but also reduce the burden on the patient's heart. Gong et al. demonstrated that high-quality nursing accompaniment can alleviate negative emotions in patients with malignant glioma and significantly enhance their quality of life, thereby increasing patient satisfaction with nursing care, highlighting its importance for clinical application (26). In individuals diagnosed with leukemia, the provision of high-quality nursing accompaniment can significantly enhance their psychological well-being, promote adherence to treatment regimens, and increase overall satisfaction with nursing services. Additionally, it can lead to a decrease in the rate of complications associated with the placement of peripherally inserted central catheters (27). In the report of Ding et al, high-quality nursing intervention can effectively reduce the psychological pressure of the first chemotherapy for patients with malignant tumor, ameliorate the psychological burden of patients, relieve patients’ anxiety and fear, thus improve the chemotherapy effect, and contribute to improve their quality of life (28). In our research, it was observed that patients receiving high-quality care exhibited significantly elevated GQOLI-74 scores across all dimensions when compared to the control group (*P* < 0.05). This finding suggests that high-quality care not only enhances the quality of life for patients with AMI but also positively influences their adherence to prescribed medications. Acute myocardial infarction requires long-term or lifelong medication, especially in elderly patients, who may have deteriorated physical function and memory, and have a low sense of self-management. This all of which will greatly reduce medication adherence and therapeutic efficacy, leading to recurrence of the disease, thus further reducing the quality of life and creating a vicious circle. Therefore, enhancing the medication compliance of patients is of paramount importance for the treatment of AMI in the elderly. In the report of Zuo et al., high-quality nursing interventions improve various aspects in the treatment of elderly cataract patients, such as effectively reducing intraocular pressure, ameliorating patients’ quality of life, lowering the incidence of postoperative complications, and improving patient satisfaction (17). This study is innovative because it carefully examines how high-quality nursing accompaniment interventions influence several important areas, such as sleep quality, psychological well-being, quality of life, complication rates, and patient satisfaction. This analysis confirms the effectiveness and safety of these interventions for individuals with acute myocardial infarction and presents an alternative treatment option for this patient group. Despite its valuable contributions, this study is not without limitations. First and foremost, the research was conducted at a single institution with a relatively small sample size. This limitation exposes the results to potential regional biases, which may affect the generalizability of the findings. Additionally, the high-quality nursing accompaniment intervention was implemented over a brief period of only three months. This short duration underscores the significant gap in long-term follow-up studies for patients diagnosed with AMI. Future research should consider longer-term interventions to better understand the sustained impact on patient outcomes. The effectiveness of the intervention could have been impacted by how well the nursing staff complied with the measures and how receptive the patients were to them. High levels of adherence from the nursing staff, along with positive engagement from the patients, are essential for the success of any intervention. Any variations in these factors may have influenced the outcomes that were observed. Another limitation of this study is the challenge in controlling potential confounding factors. Although we made efforts to consider various influences, there may have been some factors that were not sufficiently accounted for. For example, the economic status of patients and the presence of social support systems could significantly affect the effectiveness of nursing interventions. These elements are recognized to impact patient outcomes and warrant more comprehensive examination in future research. It is crucial to highlight that the researchers who analyzed the data were aware of the group assignments, which raises concerns about potential bias in the analysis due to the absence of blinding in this study. To improve the objectivity and reliability of results in future studies, it is recommended that blinding procedures be implemented. We recognize that the available comparative data on cardiac complications may be limited, primarily due to a lack of information regarding the baseline similarities between the two groups. While we did not gather detailed information on cardiovascular risk factors—such as tobacco use, diabetes, and blood pressure—or specific cardiac data like ejection fraction and related comorbidities such as cardiac insufficiency for each patient, we understand that these factors could greatly affect the outcomes. Therefore, future studies should aim to incorporate these variables to better manage potential confounding effects and to enhance our understanding of the intervention's overall impact.

## 5 Conclusion

In summary, the implementation of a high-quality nursing accompaniment model demonstrates substantial potential in improving sleep quality among individuals affected by AMI. This model not only enhances overall quality of life but also increases nursing satisfaction and reduces the incidence of complications. As such, this model merits further adoption and promotion within clinical settings.

## Data Availability

The original contributions presented in the study are included in the article/Supplementary Material, further inquiries can be directed to the corresponding authors.
